# 4-{[3-(4-Hydroxy­benzyl­ideneamino)-2,2-dimethyl­propyl]iminiomethyl}phenolate dihydrate

**DOI:** 10.1107/S1600536809013804

**Published:** 2009-04-18

**Authors:** Reza Kia, Hoong-Kun Fun, Hadi Kargar

**Affiliations:** a X-ray Crystallography Unit, School of Physics, Universiti Sains Malaysia, 11800 USM, Penang, Malaysia; b Department of Chemistry, School of Science, Payame Noor University (PNU), Ardakan, Yazd, Iran

## Abstract

The asymmetric unit of the title compound, C_19_H_22_N_2_O_2_·2H_2_O, comprises a zwitterionic form of the Schiff base compound and two water mol­ecules of crystallization. Inter­molecular N—H⋯O, C—H⋯O and O—H⋯N hydrogen bonds involving one of the water mol­ecules in the asymmetric unit generate seven- and eight-membered rings, with *R*_2_^1^(7) and *R*_2_^2^(8) ring motifs, respectively. The dihedral angle beween the two aromatic rings is 86.5 (2)°. The imino and iminium groups are coplanar with the benzene rings to which they are attached, making dihedral angles (N—C—C—C) of −179.3 (5) and −179.2 (4)°, respectively. Validation software indicates the higher symmetry space group *Pnma* for this structure. However, this process ignores H atoms and the zwitterionic configuration of the main mol­ecule breaks the higher symmetry. Solution in *Pna*2_1_ provides a chemically sensible zwitterionic compound with improved residuals. In the crystal structure, mol­ecules are linked together through inter­molecular O—H⋯O, O—H⋯N, N—H⋯O and C—H⋯O inter­actions, forming a three-dimensional network. The crystal structure is further stabilized by inter­molecular C—H⋯π inter­actions.

## Related literature

For hydrogen-bond motifs, see: Bernstein *et al.* (1995 ▶). For information on Schiff base ligands and their complexes and applications, see: Calligaris & Randaccio (1987 ▶); Li *et al.* (2005 ▶); Bomfim *et al.* (2005 ▶); Glidewell *et al.* (2005 ▶, 2006 ▶); Sun *et al.* (2004 ▶). For details of the synthesis, see: Fun *et al.* (2008 ▶). For the stability of the temperature controller used for data collection, see: Cosier & Glazer (1986 ▶).
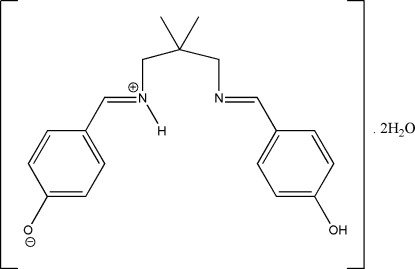


## Experimental

### 

#### Crystal data

C_19_H_22_N_2_O_2_·2H_2_O
*M* *_r_* = 346.42
Orthorhombic, 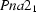

*a* = 13.0336 (4) Å
*b* = 11.5242 (3) Å
*c* = 12.4132 (4) Å
*V* = 1864.49 (10) Å^3^
*Z* = 4
Mo *K*α radiation
μ = 0.09 mm^−1^
*T* = 100 K
0.34 × 0.21 × 0.11 mm

#### Data collection

Bruker SMART APEXII CCD area-detector diffractometer
Absorption correction: multi-scan (*SADABS*; Bruker, 2005 ▶) *T*_min_ = 0.971, *T*_max_ = 0.991
10183 measured reflections
2237 independent reflections
1753 reflections with *I* > 2σ(*I*)
*R*_int_ = 0.056

#### Refinement

*R*[*F* ^2^ > 2σ(*F* ^2^)] = 0.047
*wR*(*F* ^2^) = 0.116
*S* = 1.05
2237 reflections
235 parameters
3 restraints
H atoms treated by a mixture of independent and constrained refinement
Δρ_max_ = 0.27 e Å^−3^
Δρ_min_ = −0.20 e Å^−3^

### 

Data collection: *APEX2* (Bruker, 2005 ▶); cell refinement: *APEX2*; data reduction: *SAINT* (Bruker, 2005 ▶); program(s) used to solve structure: *SHELXTL* (Sheldrick, 2008 ▶); program(s) used to refine structure: *SHELXTL*; molecular graphics: *SHELXTL*; software used to prepare material for publication: *SHELXTL* and *PLATON* (Spek, 2009 ▶).

## Supplementary Material

Crystal structure: contains datablocks global, I. DOI: 10.1107/S1600536809013804/sj2615sup1.cif

Structure factors: contains datablocks I. DOI: 10.1107/S1600536809013804/sj2615Isup2.hkl

Additional supplementary materials:  crystallographic information; 3D view; checkCIF report

## Figures and Tables

**Table 1:** Hydrogen-bond geometry (Å, °) *Cg*1 and *Cg*2 are the centroids of the C1–C6 and C12–C17 benzene rings, respectively.

*D*—H⋯*A*	*D*—H	H⋯*A*	*D*⋯*A*	*D*—H⋯*A*
O1*W*—H1*W*1⋯O1^i^	0.93	1.91	2.830 (5)	171
O1*W*—H2*W*1⋯O2^ii^	0.86	1.90	2.751 (4)	168
O2*W*—H1*W*2⋯O1*W*^iii^	0.84	2.00	2.774 (3)	153
O2*W*—H2*W*2⋯N1	0.86	2.22	2.826 (4)	127
N2—H1N2⋯O2*W*	0.95 (4)	1.90 (4)	2.832 (4)	170 (3)
O1—H1O1⋯O2^ii^	0.83 (4)	1.74 (5)	2.565 (5)	174 (5)
C17—H17*A*⋯O2*W*	0.93	2.52	3.408 (6)	161
C10—H10*B*⋯*Cg*1^iv^	0.97	2.69	3.422 (5)	133
C8—H8*A*⋯*Cg*2^v^	0.97	2.74	3.497 (5)	136

Symmetry codes: (i) 
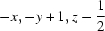
; (ii) 
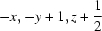
; (iii) 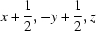; (iv) 
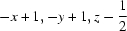
; (v) 
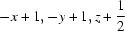
.

**Fig. 1.**
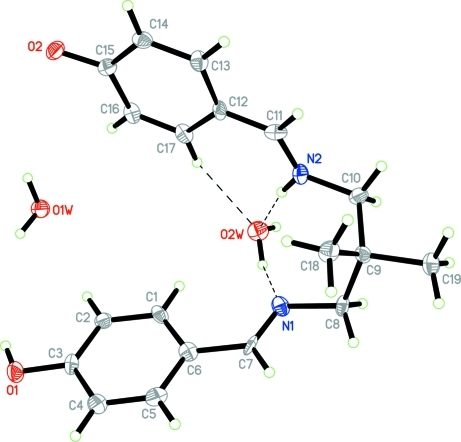
The molecular structure of the title compound with atom labels and 50% probability ellipsoids for non-H atoms. Intermolecular interactions involving the O2W water molecule are shown as dashed lines.

**Fig. 2.**
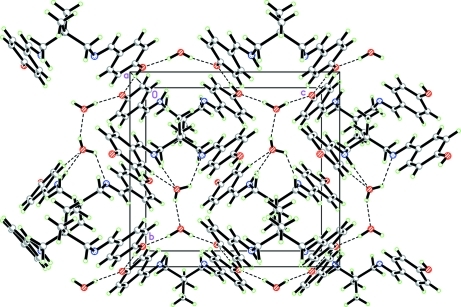
The crystal packing of the title compound, viewed down the a axis showing three-dimensional network formation through intermolecular interactions, shown as dashed lines.

## supplementary crystallographic information

### Comment

In the field of coordination chemistry, Schiff base compounds are among the most prevalent and versatile ligands.  They have received much attention due to their important roles in the development of coordination chemistry related to catalysis and enzymatic reactions, magnetism, and supramolecular architectures. In comparison to the Schiff base metal complexes, only a relatively small number of free Schiff base ligands have been structurally characterized (Calligaris & Randaccio, 1987). Structures of Schiff bases derived from substituted benzaldehydes have been reported (Li *et al.*, 2005; Bomfim *et al.*, 2005; Glidewell *et al.*, 2005, 2006; Sun *et al.*, 2004).

The asymmetric unit of the title compound, Fig. 1, comprises a zwitterionic Schiff base compound and two water molecules of crystallization. The zwitterion results from protonation of the imine N2 atom with the O2 hydroxy group deprotonated resulting in the formation of iminium and phenolate groups. Intermolecular N—H···O and C—H···O hydrogen bonds involving the O2W O atom as a bifurcated acceptor generate an *R_2_^1^(7)* ring motif (Bernstein *et al.*, 1995). Intermolecular N—H···O and O—H···N hydrogen bonds, again involving O2W, generate an *R_2_^2^(8)* ring motif. The dihedral angle beween the two phenyl rings is 86.5 (2)°. The imino and iminium groups are coplanar with the benzene rings to which they are attached making dihedral angles of -179.3 (5) and -179.2 (4)° for N1—C7—C6—C5 and N2—C11—C12—C13, respectively. This structure requires differentiating between the *Pna*21  and *Pnma* space groups to achieve a correct solution. The solution based on the higher symmetry space group (Pnma) gives a chemically nonsensible polymeric chain as the program PLATON/ADDSYM has not taken into account H atoms in determining the symmetry elements. The solution in *Pna*21 gives a chemically sensible zwitterionic compound with lower R values. In the crystal structure, the molecules are linked together through intermolecular O—H···O, O—H···N, N—H···O and C—H···O interactions, forming a three-dimensional network (Fig. 2). The crystal structure is further stabilized by intermolecular C—H···π interactions [*Cg*1 and *Cg*2 are the centroids of the C1–C6 and C12–C17 benzene rings] (Table 1).

### Experimental

The synthetic method has been described earlier (Fun *et al.*, 2008), except that 4-hydroxybenzaldehyde (2 mmol, 244 mg) and 2,2-dimethylpropane diamine (1 mmol, 102 mg) were used. Single crystals suitable for X-ray diffraction were obtained by evaporation of an ethanol solution at room temperature.

### Refinement

The H atom of the hydroxy group was located from the difference Fourier map and refined freely. The H atoms of O2W were located from the difference Fourier map and constrained to refine with the carrier atom with U_iso_(H) = 1.5U_eq_(O) with distance restraint of 0.85 (1) Å. The H atoms of O1W were located from the difference Fourier map and constrained to refine with the carrier atom with U_iso_(H) = 1.5U_eq_(O). Other N-bound and O-bound H atoms were located from the difference Fourier map and refined freely, see Table 1. The rest of the H atoms were positioned geometrically and refined using a riding model with C—H = 0.93–0.97 Å and U_iso_(H) = 1.2U_eq_(C). In the absence of sufficient anomalous scattering, 904 Friedel pairs were merged.

### Crystal data

**Table:**

C_19_H_22_N_2_O_2_·2H_2_O	*F*(000) = 744
*M**_r_* = 346.42	*D*_x_ = 1.234 Mg m^−^^3^
Orthorhombic, *P**n**a*2_1_	Mo *K*α radiation, λ = 0.71073 Å
Hall symbol: P 2c -2n	Cell parameters from 1477 reflections
*a* = 13.0336 (4) Å	θ = 2.4–29.9°
*b* = 11.5242 (3) Å	µ = 0.09 mm^−^^1^
*c* = 12.4132 (4) Å	*T* = 100 K
*V* = 1864.49 (10) Å^3^	Block, orange
*Z* = 4	0.34 × 0.21 × 0.11 mm

### Data collection

**Table:**

Bruker SMART APEXII CCD area-detector diffractometer	2237 independent reflections
Radiation source: fine-focus sealed tube	1753 reflections with *I* > 2σ(*I*)
graphite	*R*_int_ = 0.056
φ and ω scans	θ_max_ = 27.5°, θ_min_ = 2.4°
Absorption correction: multi-scan (*SADABS*; Bruker, 2005)	*h* = −15→16
*T*_min_ = 0.971, *T*_max_ = 0.991	*k* = −14→14
10183 measured reflections	*l* = −16→7

### Refinement

**Table:**

Refinement on *F*^2^	Primary atom site location: structure-invariant direct methods
Least-squares matrix: full	Secondary atom site location: difference Fourier map
*R*[*F*^2^ > 2σ(*F*^2^)] = 0.047	Hydrogen site location: inferred from neighbouring sites
*wR*(*F*^2^) = 0.116	H atoms treated by a mixture of independent and constrained refinement
*S* = 1.05	*w* = 1/[σ^2^(*F*_o_^2^) + (0.0526*P*)^2^ + 0.5548*P*] where *P* = (*F*_o_^2^ + 2*F*_c_^2^)/3
2237 reflections	(Δ/σ)_max_ < 0.001
235 parameters	Δρ_max_ = 0.27 e Å^−^^3^
3 restraints	Δρ_min_ = −0.20 e Å^−^^3^

### Special details

**Table:**

Experimental. The crystal was placed in the cold stream of an Oxford Cyrosystems Cobra open-flow nitrogen cryostat (Cosier & Glazer, 1986) operating at 100.0 (1) K.
Geometry. All esds (except the esd in the dihedral angle between two l.s. planes) are estimated using the full covariance matrix. The cell esds are taken into account individually in the estimation of esds in distances, angles and torsion angles; correlations between esds in cell parameters are only used when they are defined by crystal symmetry. An approximate (isotropic) treatment of cell esds is used for estimating esds involving l.s. planes.
Refinement. Refinement of F^2^ against ALL reflections. The weighted R-factor wR and goodness of fit S are based on F^2^, conventional R-factors R are based on F, with F set to zero for negative F^2^. The threshold expression of F^2^ > 2sigma(F^2^) is used only for calculating R-factors(gt) etc. and is not relevant to the choice of reflections for refinement. R-factors based on F^2^ are statistically about twice as large as those based on F, and R- factors based on ALL data will be even larger.

### Fractional atomic coordinates and isotropic or equivalent isotropic displacement parameters (Å^2^)

**Table:**

	*x*	*y*	*z*	*U*_iso_*/*U*_eq_	
O1	0.0498 (3)	0.5696 (3)	1.0250 (3)	0.0254 (8)	
O2	0.0475 (3)	0.5792 (3)	0.4102 (2)	0.0217 (7)	
N1	0.5104 (3)	0.5803 (3)	0.8323 (3)	0.0192 (8)	
N2	0.5136 (3)	0.5842 (3)	0.5796 (3)	0.0186 (8)	
C1	0.2946 (4)	0.5328 (4)	0.8831 (4)	0.0181 (10)	
H1A	0.3225	0.4895	0.8270	0.022*	
C2	0.1925 (4)	0.5165 (4)	0.9112 (4)	0.0178 (10)	
H2A	0.1528	0.4627	0.8741	0.021*	
C3	0.1496 (4)	0.5802 (4)	0.9947 (4)	0.0184 (10)	
C4	0.2104 (4)	0.6615 (4)	1.0498 (4)	0.0221 (10)	
H4A	0.1828	0.7050	1.1059	0.026*	
C5	0.3115 (4)	0.6764 (4)	1.0197 (4)	0.0211 (10)	
H5A	0.3510	0.7310	1.0560	0.025*	
C6	0.3563 (4)	0.6130 (4)	0.9374 (4)	0.0181 (10)	
C7	0.4645 (4)	0.6339 (4)	0.9073 (4)	0.0186 (10)	
H7A	0.5010	0.6896	0.9457	0.022*	
C8	0.6178 (4)	0.6143 (4)	0.8131 (4)	0.0172 (9)	
H8A	0.6594	0.5447	0.8092	0.021*	
H8B	0.6416	0.6593	0.8742	0.021*	
C9	0.63477 (18)	0.6854 (2)	0.7101 (5)	0.0166 (5)	
C10	0.6196 (4)	0.6143 (4)	0.6059 (4)	0.0207 (10)	
H10A	0.6481	0.6580	0.5463	0.025*	
H10B	0.6588	0.5431	0.6122	0.025*	
C11	0.4577 (4)	0.6396 (3)	0.5096 (3)	0.0173 (9)	
H11A	0.4891	0.7009	0.4737	0.021*	
C12	0.3548 (4)	0.6168 (4)	0.4823 (4)	0.0160 (9)	
C13	0.3087 (4)	0.6874 (4)	0.4023 (4)	0.0186 (10)	
H13A	0.3476	0.7432	0.3668	0.022*	
C14	0.2061 (4)	0.6736 (4)	0.3767 (4)	0.0183 (9)	
H14A	0.1768	0.7206	0.3241	0.022*	
C15	0.1448 (4)	0.5894 (4)	0.4291 (4)	0.0172 (9)	
C16	0.1949 (4)	0.5154 (3)	0.5056 (4)	0.0182 (10)	
H16A	0.1574	0.4564	0.5382	0.022*	
C17	0.2949 (4)	0.5287 (3)	0.5318 (4)	0.0181 (10)	
H17A	0.3246	0.4798	0.5826	0.022*	
C18	0.56782 (19)	0.7944 (2)	0.7087 (5)	0.0209 (6)	
H18A	0.5770	0.8364	0.7748	0.031*	
H18B	0.4971	0.7725	0.7014	0.031*	
H18C	0.5873	0.8426	0.6491	0.031*	
C19	0.74958 (18)	0.7180 (2)	0.7099 (6)	0.0230 (6)	
H19A	0.7659	0.7590	0.7750	0.034*	
H19B	0.7640	0.7666	0.6490	0.034*	
H19C	0.7903	0.6487	0.7057	0.034*	
O1W	0.02979 (14)	0.33777 (16)	0.7189 (3)	0.0223 (5)	
H1W1	0.0021	0.3754	0.6592	0.033*	
H2W1	0.0012	0.3713	0.7731	0.033*	
O2W	0.45692 (15)	0.38775 (16)	0.7030 (3)	0.0247 (5)	
H1W2	0.4922	0.3279	0.6916	0.037*	
H2W2	0.4735	0.4056	0.7681	0.037*	
H1N2	0.487 (3)	0.520 (3)	0.618 (3)	0.020 (9)*	
H1O1	0.020 (3)	0.524 (4)	0.984 (4)	0.040 (14)*	

### Atomic displacement parameters (Å^2^)

**Table:**

	*U*^11^	*U*^22^	*U*^33^	*U*^12^	*U*^13^	*U*^23^
O1	0.0152 (16)	0.0329 (16)	0.0281 (17)	−0.0051 (14)	0.0031 (13)	−0.0086 (14)
O2	0.0171 (16)	0.0261 (15)	0.0219 (16)	−0.0001 (13)	−0.0042 (13)	0.0062 (12)
N1	0.020 (2)	0.0172 (18)	0.0207 (18)	−0.0025 (17)	0.0044 (15)	0.0045 (16)
N2	0.0145 (19)	0.0181 (18)	0.0231 (19)	−0.0025 (16)	0.0018 (15)	−0.0003 (15)
C1	0.019 (2)	0.019 (2)	0.016 (2)	0.001 (2)	0.0061 (19)	0.0063 (18)
C2	0.018 (3)	0.020 (2)	0.015 (2)	−0.0032 (19)	−0.0010 (19)	0.0004 (17)
C3	0.012 (2)	0.0189 (19)	0.025 (2)	−0.0004 (17)	0.0006 (17)	0.0025 (18)
C4	0.022 (2)	0.0227 (19)	0.022 (2)	0.0024 (19)	0.0011 (19)	−0.0022 (18)
C5	0.026 (3)	0.0182 (18)	0.019 (2)	−0.0060 (19)	−0.004 (2)	0.0028 (18)
C6	0.021 (2)	0.0139 (18)	0.019 (2)	0.0006 (19)	0.0014 (18)	0.0042 (19)
C7	0.0090 (19)	0.024 (2)	0.023 (2)	−0.0051 (17)	−0.0087 (18)	0.0076 (18)
C8	0.010 (2)	0.017 (2)	0.025 (2)	0.0022 (19)	−0.0017 (18)	0.004 (2)
C9	0.0150 (11)	0.0190 (11)	0.0157 (13)	−0.0035 (10)	0.002 (2)	−0.004 (2)
C10	0.021 (2)	0.025 (2)	0.016 (2)	−0.004 (2)	−0.0033 (19)	0.000 (2)
C11	0.029 (2)	0.0100 (16)	0.013 (2)	0.0029 (18)	−0.0015 (18)	−0.0009 (15)
C12	0.012 (2)	0.0166 (18)	0.020 (2)	0.0025 (18)	0.0026 (17)	−0.0080 (19)
C13	0.018 (2)	0.0142 (17)	0.024 (2)	−0.0012 (17)	0.0025 (19)	0.0039 (17)
C14	0.023 (2)	0.0161 (18)	0.016 (2)	0.0023 (18)	0.0008 (19)	0.0027 (17)
C15	0.021 (2)	0.0144 (16)	0.017 (2)	0.0029 (18)	−0.0014 (18)	−0.0027 (17)
C16	0.018 (3)	0.0117 (18)	0.025 (3)	0.0003 (18)	0.0044 (19)	0.0002 (18)
C17	0.021 (2)	0.0116 (17)	0.022 (2)	0.0041 (18)	0.0054 (19)	0.0051 (18)
C18	0.0220 (12)	0.0170 (12)	0.0238 (15)	−0.0011 (10)	−0.001 (3)	−0.006 (2)
C19	0.0181 (12)	0.0244 (13)	0.0265 (16)	−0.0061 (11)	0.002 (3)	0.001 (3)
O1W	0.0244 (10)	0.0209 (9)	0.0216 (13)	0.0046 (8)	−0.0003 (16)	−0.0010 (13)
O2W	0.0286 (11)	0.0201 (9)	0.0255 (14)	0.0002 (8)	−0.0010 (18)	−0.0041 (16)

### Geometric parameters (Å, °)

**Table:**

O1—C3	1.359 (6)	C9—C10	1.543 (7)
O1—H1O1	0.83 (4)	C10—H10A	0.9700
O2—C15	1.295 (6)	C10—H10B	0.9700
N1—C7	1.267 (6)	C11—C12	1.407 (7)
N1—C8	1.473 (6)	C11—H11A	0.9300
N2—C11	1.302 (6)	C12—C13	1.418 (6)
N2—C10	1.461 (6)	C12—C17	1.421 (6)
N2—H1N2	0.95 (4)	C13—C14	1.384 (8)
C1—C2	1.388 (7)	C13—H13A	0.9300
C1—C6	1.398 (7)	C14—C15	1.415 (7)
C1—H1A	0.9300	C14—H14A	0.9300
C2—C3	1.387 (6)	C15—C16	1.434 (6)
C2—H2A	0.9300	C16—C17	1.352 (7)
C3—C4	1.405 (7)	C16—H16A	0.9300
C4—C5	1.380 (8)	C17—H17A	0.9300
C4—H4A	0.9300	C18—H18A	0.9600
C5—C6	1.385 (7)	C18—H18B	0.9600
C5—H5A	0.9300	C18—H18C	0.9600
C6—C7	1.480 (6)	C19—H19A	0.9600
C7—H7A	0.9300	C19—H19B	0.9600
C8—C9	1.535 (7)	C19—H19C	0.9600
C8—H8A	0.9700	O1W—H1W1	0.9309
C8—H8B	0.9700	O1W—H2W1	0.8614
C9—C18	1.529 (3)	O2W—H1W2	0.8404
C9—C19	1.543 (3)	O2W—H2W2	0.8612
			
C3—O1—H1O1	110 (4)	C9—C10—H10A	108.3
C7—N1—C8	115.9 (4)	N2—C10—H10B	108.3
C11—N2—C10	124.2 (4)	C9—C10—H10B	108.3
C11—N2—H1N2	121 (2)	H10A—C10—H10B	107.4
C10—N2—H1N2	115 (2)	N2—C11—C12	127.0 (4)
C2—C1—C6	121.3 (4)	N2—C11—H11A	116.5
C2—C1—H1A	119.4	C12—C11—H11A	116.5
C6—C1—H1A	119.4	C11—C12—C13	117.7 (4)
C3—C2—C1	120.2 (4)	C11—C12—C17	123.6 (4)
C3—C2—H2A	119.9	C13—C12—C17	118.7 (4)
C1—C2—H2A	119.9	C14—C13—C12	120.3 (4)
O1—C3—C2	123.0 (4)	C14—C13—H13A	119.8
O1—C3—C4	117.7 (4)	C12—C13—H13A	119.8
C2—C3—C4	119.3 (4)	C13—C14—C15	121.3 (4)
C5—C4—C3	119.3 (5)	C13—C14—H14A	119.4
C5—C4—H4A	120.3	C15—C14—H14A	119.4
C3—C4—H4A	120.3	O2—C15—C14	122.1 (4)
C4—C5—C6	122.4 (4)	O2—C15—C16	120.8 (4)
C4—C5—H5A	118.8	C14—C15—C16	117.1 (4)
C6—C5—H5A	118.8	C17—C16—C15	122.0 (4)
C5—C6—C1	117.5 (4)	C17—C16—H16A	119.0
C5—C6—C7	120.1 (4)	C15—C16—H16A	119.0
C1—C6—C7	122.3 (4)	C16—C17—C12	120.5 (4)
N1—C7—C6	123.8 (4)	C16—C17—H17A	119.8
N1—C7—H7A	118.1	C12—C17—H17A	119.8
C6—C7—H7A	118.1	C9—C18—H18A	109.5
N1—C8—C9	114.5 (3)	C9—C18—H18B	109.5
N1—C8—H8A	108.6	H18A—C18—H18B	109.5
C9—C8—H8A	108.6	C9—C18—H18C	109.5
N1—C8—H8B	108.6	H18A—C18—H18C	109.5
C9—C8—H8B	108.6	H18B—C18—H18C	109.5
H8A—C8—H8B	107.6	C9—C19—H19A	109.5
C18—C9—C8	111.4 (4)	C9—C19—H19B	109.5
C18—C9—C19	110.7 (2)	H19A—C19—H19B	109.5
C8—C9—C19	105.7 (4)	C9—C19—H19C	109.5
C18—C9—C10	110.7 (4)	H19A—C19—H19C	109.5
C8—C9—C10	113.3 (2)	H19B—C19—H19C	109.5
C19—C9—C10	104.6 (4)	H1W1—O1W—H2W1	104.2
N2—C10—C9	115.8 (4)	H1W2—O2W—H2W2	102.5
N2—C10—H10A	108.3		
			
C6—C1—C2—C3	−0.1 (7)	C11—N2—C10—C9	99.7 (5)
C1—C2—C3—O1	178.9 (4)	C18—C9—C10—N2	−53.5 (5)
C1—C2—C3—C4	0.4 (7)	C8—C9—C10—N2	72.5 (4)
O1—C3—C4—C5	−178.6 (4)	C19—C9—C10—N2	−172.8 (4)
C2—C3—C4—C5	0.0 (7)	C10—N2—C11—C12	−178.4 (4)
C3—C4—C5—C6	−0.7 (7)	N2—C11—C12—C13	−179.2 (4)
C4—C5—C6—C1	0.9 (7)	N2—C11—C12—C17	1.9 (7)
C4—C5—C6—C7	179.0 (4)	C11—C12—C13—C14	−176.2 (4)
C2—C1—C6—C5	−0.6 (7)	C17—C12—C13—C14	2.8 (7)
C2—C1—C6—C7	−178.6 (4)	C12—C13—C14—C15	−0.1 (7)
C8—N1—C7—C6	179.1 (4)	C13—C14—C15—O2	176.4 (4)
C5—C6—C7—N1	−179.3 (4)	C13—C14—C15—C16	−3.1 (7)
C1—C6—C7—N1	−1.3 (7)	O2—C15—C16—C17	−175.9 (5)
C7—N1—C8—C9	−106.9 (4)	C14—C15—C16—C17	3.6 (7)
N1—C8—C9—C18	56.0 (4)	C15—C16—C17—C12	−1.0 (7)
N1—C8—C9—C19	176.4 (4)	C11—C12—C17—C16	176.6 (4)
N1—C8—C9—C10	−69.6 (4)	C13—C12—C17—C16	−2.3 (7)

### Hydrogen-bond geometry (Å, °)

**Table:**

*D*—H···*A*	*D*—H	H···*A*	*D*···*A*	*D*—H···*A*
O1W—H1W1···O1^i^	0.93	1.91	2.830 (5)	171
O1W—H2W1···O2^ii^	0.86	1.90	2.751 (4)	168
O2W—H1W2···O1W^iii^	0.84	2.00	2.774 (3)	153
O2W—H2W2···N1	0.86	2.22	2.826 (4)	127
N2—H1N2···O2W	0.95 (4)	1.90 (4)	2.832 (4)	170 (3)
O1—H1O1···O2^ii^	0.83 (4)	1.74 (5)	2.565 (5)	174 (5)
C17—H17A···O2W	0.93	2.52	3.408 (6)	161
C10—H10B···Cg1^iv^	0.97	2.69	3.422 (5)	133
C8—H8A···Cg2^v^	0.97	2.74	3.497 (5)	136

Symmetry codes: (i) −*x*, −*y*+1, *z*−1/2; (ii) −*x*, −*y*+1, *z*+1/2; (iii) *x*+1/2, −*y*+1/2, *z*; (iv) −*x*+1, −*y*+1, *z*−1/2; (v) −*x*+1, −*y*+1, *z*+1/2.
